# Cytomegalovirus as a potential trigger for systemic lupus erythematosus: a case report

**DOI:** 10.1186/s13104-015-1520-2

**Published:** 2015-09-28

**Authors:** Susumu Yamazaki, Amane Endo, Takashi Iso, Shinpei Abe, You Aoyagi, Mitsuyoshi Suzuki, Toru Fujii, Hidenori Haruna, Yoshikazu Ohtsuka, Toshiaki Shimizu

**Affiliations:** Department of Pediatrics and Adolescent Medicine, Juntendo University Graduate School of Medicine, 2-1-1, Hongo, Bunkyo-ku, Tokyo, 113-8421 Japan

**Keywords:** Systemic lupus erythematosus, Cytomegalovirus infection, Ganciclovir

## Abstract

**Background:**

The role of cytomegalovirus infection in triggering systemic lupus erythematosus remains a subject of debate. Here, we present a case of childhood systemic lupus erythematosus with concomitant cytomegalovirus infection, which sheds light on the relationship between these conditions and their treatment in pediatric patients.

**Case presentation:**

A 12-year-old Japanese girl with no history of systemic illness was diagnosed with systemic lupus erythematosus and concomitant primary cytomegalovirus infection. Her anti-cytomegalovirus immunoglobulin G antibodies were elevated during diagnosis and treatment. Further, the patient’s cytomegalovirus pp65 antigenemia level was slightly elevated (1 cell per 5 × 10^4^ cells). Treatment included the administration of ganciclovir, prednisolone, methylprednisolone, and cyclophosphamide, none of which prompted adverse effects. The patient was in good condition at the most recent follow-up.

**Conclusion:**

Ganciclovir treatment is not completely safe, and there are no clinical guidelines regarding its use in patients with systemic lupus erythematosus triggered by cytomegalovirus infection. Our experience with this case suggests that the decision to administer ganciclovir treatment in pediatric cases should be guided by a variety of factors in addition to the cytomegalovirus antigenemia level. These factors include lymphopenia, renal biopsy results, and cytomegalovirus DNA levels detected by polymerase chain reaction. The details of our patient’s presentation and treatment should prove illustrative to other clinicians who face similar cases.

## Background

In recent decades, many researchers have focused on the role of viral infection in the etiopathogenesis of systemic lupus erythematosus (SLE); cytomegalovirus (CMV) is considered to be one of the viruses that may trigger SLE [1].

Although there have been several reports that CMV infection may trigger the onset of SLE, its role in triggering SLE remains a subject of debate [2–4]. Because similar features are shared by both diseases, it is difficult to determine whether CMV is the primary infection, and whether clinical manifestations are due to CMV infection or exacerbation of SLE. Accordingly, clinical manifestations may actually represent a secondary exacerbation of CMV infection by undiagnosed SLE. Furthermore, although it has been reported that CMV infection may trigger SLE, there are no clinical guidelines regarding the treatment of patients with SLE and concomitant primary CMV infection.

Here, we describe the case of a 12-year-old girl whose anti-CMV immunoglobulin G (IgG) antibodies were elevated when she was first diagnosed with SLE.

## Case presentation

A 12-year-old Japanese girl was referred to our department with cervical lymphadenitis and liver dysfunction, which she had experienced for 1 month prior to presentation. Physical examination showed no abnormalities except for slight swelling of the right cervical lymph nodes. Initial laboratory results revealed a normal complete blood cell count, but an elevated aspartate aminotransferase (AST) level (96 IU/L), an elevated alanine aminotransferase (ALT) level (241 IU/L), and anti-CMV immunoglobulin M (IgM) antibodies (3.08) (detected by enzyme immunoassay [EIA]; positive titer, >1.21). Based on these results, we first diagnosed transient liver dysfunction due to CMV infection. In general, most cases of CMV infection are self-limited; therefore, our patient was discharged to her home and no additional medications were provided at that time. Although we advised the patient to receive subsequent consultation a week later to follow-up on her liver dysfunction, she did not do so.

Two months after our examination, the patient returned to our hospital with recurrent fever, butterfly eruption, and palmar erythema. On examination, her vital signs were normal without low-grade fever (37.7 °C). Her neurologic exam also was normal. Nikolsky’s sign was negative, there were no mucosal erosions or blisters, and Raynaud phenomenon was absent. Abdominal examination did not reveal hepatosplenomegaly. We suspected SLE, and admitted the patient to the hospital.

Laboratory tests on admission were significant for presence of leucopenia and thrombocytopenia (white blood cell count, 5000/μL; neutrophil count, 4075/μL; lymphocyte count, 650/μL; hemoglobin level, 12.3 g/dL; platelet count, 12.3 × 10^4^/μL). Her international normalized ratio, partial thromboplastin time, and C-reactive protein level were within normal limits, and her serum creatinine level was 0.77 mg/dL. Liver dysfunction showed slight improvement compared with the assessment performed 2 months prior (AST level, 76 IU/L; ALT level, 77 IU/L). Antinuclear antibody was positive in 1/640 titers, which showed a speckled and homogeneous pattern. Double-stranded DNA (ds-DNA) level was elevated (119 IU/mL), and complement levels were low [C3, 43 mg/dL (reference range, 86–160 mg/dL); C4, 6 mg/dL (reference range, 17–45 mg/dL)]. Anticardiolipin antibody and lupus anticoagulant were negative. Anti-Ro, anti-La, anti-Sm, and anticentromere antibodies were also negative. Anti-CMV IgM antibodies were elevated (4.16), as were anti-CMV IgG antibodies (9.1) (EIA; positive titer, >4.0). CMV pp65 antigenemia level was modestly elevated (1 cell per 5 × 10^4^ cells), and CMV DNA was detected in her urine, but not in her blood, by polymerase chain reaction (PCR). Her twenty-four-hour urinary protein level was 2.4 g/d. Renal biopsy was performed, which revealed class III lupus nephritis, as assessed according to the International Society of Nephrology/Renal Pathology Society classification.

**Fig. 1:**
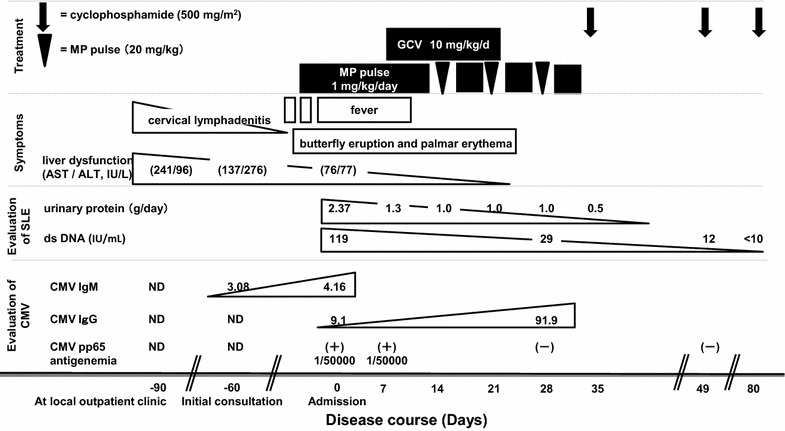
Disease course in the reported case. The course of lupus progression can be seen to correlate with anti-cytomegalovirus immunoglobulin G levels and, subsequently, with increasing immunoglobulin G levels. *ALT* alanine aminotransferase, *AST* aspartate aminotransferase, *CMV* cytomegalovirus, *ds DNA* double-stranded DNA, *GCV* ganciclovir, *IgG* immunoglobulin G, *IgM* immunoglobulin M, *MP* methylprednisolone, *ND* not documented

The patient was diagnosed with SLE and concomitant primary CMV infection. We began treatment with 1 mg kg^−1^ day^−1^ prednisolone for the SLE and 10 mg kg^−1^ day^−1^ ganciclovir for the CMV infection, until tests for CMV DNA were confirmed to be negative in her blood and urine, which occurred after 10 days of treatment. Subsequently, the patient was administered 20 mg kg^−1^ day^−1^ methylprednisolone 3 days per week, followed by 0.8 mg kg^−1^ day^−1^ prednisolone; she repeated this therapy three times. These treatments were effective for symptoms of SLE: fever improved within a week after admission, while skin lesions and liver dysfunction improved within a week after methylprednisolone treatment. Ganciclovir was administered for a total of 14 days. On day 36, the patient also was administered 500 mg/m^2^ cyclophosphamide according to American College of Rheumatology guidelines. She was discharged after 6 weeks. At discharge, her ds-DNA level had improved to 29 IU/mL, her urinary protein level was 0.5 g/d, her CMV IgG level was elevated (91.9), and her CMV pp65 antigenemia level was normal. After discharge, the patient received the remaining cyclophosphamide therapy five times at our ambulatory clinic. Currently, the patient has a good condition with normal ds-DNA and urinary protein levels. There were no adverse effects of the drugs administered during this course of treatment (Fig. 1).

## Conclusions

Two concern warrant discussion regarding this patient. The first is whether the CMV infection actually caused SLE. The second is whether this patient actually required ganciclovir treatment.

In recent decades, many studies have focused on the role of viral infection in the etiopathogenesis of SLE, which is often called the viral hypothesis [5]. Although there is no evidence that CMV leads to SLE or predisposes patients to SLE, previous reports have suggested that CMV pp65 subfragment peptide elicits the production of antibodies that cross-react with nuclear proteins and are pathogenic in genetically susceptible individuals [6, 7]. In addition, there have been several reports that CMV infection might trigger the onset of SLE [2, 3]. However, the patients in these cases were diagnosed according to positive anti-CMV IgG or anti-CMV IgM antibodies, and their anti-CMV IgG antibodies were already elevated when they were diagnosed with SLE. It is difficult to determine whether CMV is the primary infection because primary CMV infection may produce only mild catarrh or nonspecific symptoms, and it has been reported that antinuclear antibodies appear before SLE develops [8]. Therefore, in those previous cases [2, 3], the clinical manifestations may have been a secondary exacerbation of CMV infection by undiagnosed SLE. In our patient, anti-CMV IgG antibodies were elevated during the diagnosis and treatment of SLE. Therefore, we believe that this case report offers better evidence than previous reports for interpreting CMV infection as a potential trigger for SLE.

Since CMV infection in lupus is known to be a potential trigger for macrophage activation syndrome, the administration of ganciclovir is considered to be the standard treatment. In general, however, current use of this drug in clinical practice is limited due to dose-dependent toxicity. Ganciclovir is known to have such adverse effects as myelosuppression, irreversible suppression of fertility, and liver or kidney injury. For example, Abdallah et al. reported a case of ganciclovir-induced acute liver injury in an 18-year-old woman with lupus nephritis [9].

Although the use of ganciclovir is not entirely safe, there are no clinical guidelines regarding its use in patients with SLE triggered by CMV infection, and evidence concerning pediatric cases is especially limited. In adults, Arai et al. suggested ganciclovir treatment for CMV infection in patients with diffuse parenchymal lung disease and a high CMV pp65 antigenemia level (>7.5 cells per 5 × 10^4^ cells) [10]. Takizawa et al. studied CMV infection in patients with rheumatic disease, and presented some clues regarding when to start antiviral therapy. They recommended that older ages (>59.3 years) and the presence of lymphopenia may indicate that CMV therapy should be initiated. In addition, a high CMV pp65 antigenemia level (>2.8 cells per 5 × 10^4^ cells) was one of the risk factors for a fatal outcome [11]. In our case, we decided to administer ganciclovir despite the patient’s low CMV pp65 antigenemia level because she had lymphopenia, CMV DNA was detected by PCR, and her renal biopsy grade required immunosuppressive treatment, which may worsen infection.

In conclusion, we have described the case of a patient with SLE whose anti-CMV IgG antibodies were elevated at diagnosis. We believe this rare report proves that primary CMV infection occurred simultaneously with the development of SLE symptoms. When deciding whether to administer ganciclovir treatment in similar pediatric cases, one should be careful to assess not only the CMV antigenemia level, but also other findings, such as lymphopenia, renal biopsy results, and CMV DNA level detected by PCR.

## Abbreviations

ALT: alanine aminotransferase
AST: aspartate aminotransferase
CMV: cytomegalovirus
ds-DNA: double-stranded DNA
EIA: enzyme immunoassay
IgG: immunoglobulin G
IgM: immunoglobulin M
PCR: polymerase chain reaction
SLE: systemic lupus erythematosus
